# Supplementary material for: The prognostic and clinicopathological significance of SLC7A11 in human cancers: a systematic review and meta-analysis
Source: PeerJ. 2023 Feb 27;11:e14931. doi: 10.7717/peerj.14931 (PMC9979827; doi:10.7717/peerj.14931)
Supplement: Supplemental Information 1 — The rationale of our systematic review and meta-analysis. [file peerj-11-14931-s001.docx]

1. **The rationale for conducting the systematic review / meta-analysis.**

It is of great value to identify factors closely related to cancer initiation and progression that can be used as effective biomarkers for early diagnosis, prognosis evaluation, and treatment guidance in cancer patients. A number of published studies have reported the survival and clinicopathological significance of solute carrier family 7 member 11 (SLC7A11), but the results were still controversial. Therefore, we performed this systematic review and meta-analysis to explore the prognostic value of SLC7A11.

1. **The contribution that it makes to knowledge in light of previously published related reports, including other meta-analyses and systematic reviews.**

This study aimed at review the prognostic value of SLC7A11 systematically. It could contribute to the knowledge of the survival and clinicopathological significance of SLC7A11. No systematic review and meta-analysis has been published on this issue. Therefore, this systematic review and meta-analysis is a new study on this issue.
